# Leukocyte Telomere Length Is Not Reduced in Children and Adults with Cystic Fibrosis but Associates with Clinical Characteristics—A Cross-Sectional Study

**DOI:** 10.3390/jcm10040590

**Published:** 2021-02-04

**Authors:** Aleksandra Glapa-Nowak, Shivaprakash Jagalur Mutt, Aleksandra Lisowska, Ewa Sapiejka, Joanna Goździk-Spychalska, Mirosława Wieczorek-Filipiak, Sławomira Drzymała-Czyż, Jan Krzysztof Nowak, Olaf Thalmann, Karl-Heinz Herzig, Jarosław Walkowiak

**Affiliations:** 1 Department of Paediatric Gastroenterology and Metabolic Diseases, Poznań University of Medical Sciences, 60-572 Poznań, Poland; glapa@ump.edu.pl (A.G.-N.); alisowska@ump.edu.pl (A.L.); filipiaki@poczta.onet.pl (M.W.-F.); drzymala@ump.edu.pl (S.D.-C.); jannowak@ump.edu.pl (J.K.N.); othalmann@ump.edu.pl (O.T.); karl-heinz.herzig@oulu.fi (K.-H.H.); 2 Research Unit of Biomedicine, University of Oulu, 90220 Oulu, Finland; shivaprakash.jagalur@oulu.fi; 3 Medical Research Center, University of Oulu, Oulu University Hospital, 90220 Oulu, Finland; 4 The Specialist Centre for Medical Care of Mother and Child, 80-308 Gdańsk, Poland; e.sapiejka@wp.pl; 5 Department of Pulmonology, Allergology and Respiratory Oncology, Poznan University of Medical Sciences, 60-569 Poznań, Poland; jogoz@ump.edu.pl; 6 Department of Bromatology, Poznan University of Medical Sciences, 60-354 Poznań, Poland

**Keywords:** CFTR, dried blood spots, inhaled corticosteroids, *Pseudomonas aeruginosa*, pancreatic insufficiency

## Abstract

We hypothezied that telomere length is considerably altered in cystic fibrosis (CF) patients compared to healthy subjects (HS), and that leukocyte telomere length variation reflects the severity of CF. Relative telomere length (RTL) was assessed by qPCR in 70 children aged 5–10 (34 CF; 36 HS) and 114 adults aged 18–45 (53 CF; 61 HS). Telomere length was similar in CF and HS (median (interquartile range): 0.799 (0.686–0.950) vs. 0.831 (0.707–0.986); *p* = 0.5283) both in children and adults. In adults, women had longer telomeres than men (0.805 (0.715–0.931) vs. 0.703 (0.574–0.790); *p* = 0.0002). Patients treated with inhaled corticosteroids had a shorter RTL compared to those without steroid therapy (0.765 (0.664–0.910) vs. 0.943 (0.813–1.191); *p* = 0.0007) and this finding remained significant after adjusting for gender, age, BMI, and child/adult status (*p* = 0.0003). Shorter telomeres were independently associated with the presence of comorbidities (0.763 (0.643–0.905) vs. 0.950 (0.783–1.130); *p* = 0.0006) and antibiotic treatment at the moment of blood sampling (0.762 (0.648–0.908) vs. 0.832 (0.748–1.129); *p* = 0.0172). RTL correlated with number of multiple-day hospitalizations (rho = −0.251; *p* = 0.0239), as well as number of hospitalization days (rho = −0.279; *p* = 0.0113). Leukocyte RTL in children and adults with CF was not shorter than in healthy controls, and did not seem to have any potential as a predictor of CF survival. However, it inversely associated with the investigated clinical characteristics.

## 1. Introduction

Telomeres in humans are the repetitive TTAGGG sequences at the end of chromosomes that protect them from chromosome fusion and shortening whenever cells divide [1]. Furthermore, telomeres play a crucial role in maintaining genome stability, by protecting against DNA damage signals [2,3]. Telomere length depends on a complex interplay of genetic and epigenetic changes in telomere-associated proteins, telomerase, and epigenetic enzymes. Telomere degradation has been associated with lifestyle changes and various diseases, including cardiovascular dysfunction, diabetes mellitus, acute kidney injury, and cancer [4,5]. More specifically, in chronic lymphocytic leukemia, telomere length of less than 5000 bp was associated with poor treatment-free, and overall, survival [6]. Furthermore, telomere length measured in pediatric malignancies correlated with disease outcome and treatment progress [7]. Lastly, several studies focusing on chronic liver disease and diabetes reported that organ dysfunction is reflected in a rapid decline in telomere length [8,9]. These studies demonstrated a correlation of various diseases with telomere length variation. Therefore, we were interested in studying telomere length in cystic fibrosis (CF).

Despite recent progress in CF studies, it still remains one of the most common life-threatening genetic diseases in Europe, with recurrent respiratory tract infections as a major contributor to respiratory dysfunction [10]. CF results from mutations in the gene coding the transmembrane conductance regulator (CFTR), which leads to an increase in mucus viscosity and, consequently, organ damage. To date, there are over 2100 mutations identified in *CFTR* gene, of which the most common leads to the deletion of phenylalanine F508del, and accounts for ~70% in Europe and ~55% in Poland [11]. *CFTR* mutations are categorized into six classes, which combine *CFTR* defect and clinical features [12]. Traditionally, class I mutations are stop-codon mutations, and along with class II are considered to lead to severe phenotype [12]. Respiratory dysfunction is potentiated by pancreatic insufficiency and malnutrition [13,14]. Chronic inflammation and fat-soluble vitamin deficiencies exacerbate oxidative stress, which is a major contributor to telomere shortening [15].

In the present study we investigated telomere length in leukocyte DNA isolated from the dried capillary blood spots of patients with CF and healthy controls. By recruiting a narrow-age range cohort of children we attempted to limit the impact of confounding factors related to lifestyle and disease severity. Furthermore, we hypothesized that telomere length alteration would correlate with disease progression, and thus we applied the same methodology in adults with CF and healthy individuals. We further tested if telomere length is associated with hospitalizations and other clinical characteristics of CF, as these parameters can be indicative of the severity of the disease.

## 2. Materials and Methods

### 2.1. Patients

The pediatric cohort included 34 children with CF and 36 HS aged 5–10 years (Table 1) recruited at remission during routine checkups (one-day visits between 12/2017 and 06/2018). Study participants were enrolled in the Specialist Centre for Medical Care of Mother and Child, Gdańsk, Poland, and in the Department of Pediatric Gastroenterology and Metabolic Diseases, Poznan, Poland. Patients were appointed independently to the research schedule. The inclusion criteria for both groups of children were: age 5–10 years, gestational age ≥36 weeks, and birth weight >2500 g. Exclusion criteria for controls were: birth defects, congenital metabolic disorders, significant perinatal complications, chronic systemic diseases, and acute infections in the preceding month (diarrhea, respiratory infections). Healthy children were recruited in a pediatric general practice, during routine health checks. CF children were infection-free at the time of blood drawing (C-reactive protein levels <0.2 mg/dL; complete blood count with no significant deviations from the reference range). Standardized body weight, height, and body mass index (BMI) were calculated from reference Polish population [16].

The adult cohort included 53 adults with CF, and 61 HS aged 18–45 years (Table 1). All patients were recruited during routine checkups and hospital admissions in the Specialist Centre for Medical Care of Mother and Child, Gdańsk, Poland, and in the Department of Pulmonology, Allergology, and Respiratory Oncology Poznan, Poland. The inclusion criterion for CF was a willingness to participate in the study. Exclusion criteria comprised: organ transplantation, chronic systemic CF non-related disease, pregnancy, and smoking. The inclusion criteria for the control group was BMI within the reference range. Criteria for the exclusion of controls involved: significant health problems (chronic systemic disease or acute infections in the preceding month), smoking, and pregnancy.

The presence of homozygous or compound heterozygous class I or II mutations in CF patients was considered a severe genotype (Table S1 (Supplementary Materials)) [12]. Patients were divided into pancreatic sufficient and insufficient on the basis of elastase-1 concentration in stools (ELISA; Schebo Biotech, Giessen, Germany) [17,18,19]. Patient’s FEV_1_% was collected from the medical records (value closest to blood collection). CF-related liver disease and diabetes were diagnosed according to the EuroCareCF guidelines [20]. *Pseudomonas aeruginosa* infection was determined by both intermittent and chronic culture-validated colonization. The data on comorbidities, as well as on hospitalizations and pulmonary exacerbations for the past 5 years, were extracted from medical records. None of the enrolled patients had received CFTR modulator treatment.

The study was approved by the Bioethical Committee of Poznan University of Medical Sciences, Poland (protocol 692/17).

### 2.2. Telomere Length Assessment

Capillary blood was collected on screening cards (Eastern Business Forms Inc., Greenville, SC, USA), which were left to dry at room temperature for at least 4 h, out of direct sunlight. The genetic material from one quarter of the spot was isolated using a same-lot NucleoSpin Tissue kit, according to the manufacturer’s protocol (Macherey–Nagel, Düren, Germany) [21]. No post-extraction procedures were performed. The concentration of DNA was assessed by quantitative polymerase chain reaction (qPCR) using a serial dilution curve. Relative telomere length (RTL) was determined by the qPCR method, using primers for amplification of telomere and single copy gene from [22]. The PCR cycling conditions for the telomere primers were: 95 °C for 10 min, 2 repeats of: 15 s at 95 °C followed by 15 s at 45 °C; 40 cycles of: 15 s at 95 °C, 15 s at 60 °C, and one minute at 70 °C with signal acquisition. The following melt curve started with 15 s at 95 °C, then 71 cycles increasing by +0.5 °C (60–95 °C) for PCR product verification. The thermoprofile for the single copy gene was 95 °C for 10 min, 55 cycles of: 15 s at 95 °C, 1 min at 60 °C with signal acquisition, and melt curve starting 15 s at 95 °C, and then increasing temperature by +0.5 °C (60–95 °C) for PCR product verification [23,24]. To reflect the nature of the DNA being analyzed in clinical samples, the reference DNA sample for the standard curve consisted of pooled DNA extracted from whole blood, as well as DNA extracted from each dried blood sample, as suggested by Lin et al. [25]. A two-fold serial dilution of a 6-point standard curve (dilutions ranging from 25 to 0.78 ng/μL) was included in each plate, with each standard and sample run in triplicate and no samples in the edge wells of the plates [26]. The RTL (telomere/single copy gene ratio) was calculated from the telomere and single copy gene Ct values, and compared to the reference sample (calibrator) included in each plate using the 2-ΔΔCt method. All measurements were performed by the same operator to diminish pipetting error, and samples were encoded by the operator. The z scores of RTL were calculated in relation to the mean and SD derived from the HS group. 

### 2.3. Statistical Analysis

Prior to recruitment, the sample size required was calculated based on the RTL observed in patients with severe disease compared to healthy controls. Assuming that the differences in the RTL observed between CF and HS are higher than 20% (SD = 25%), 25 subjects would be required in each group (α = 0.05; β = 0.2). The normality of the data distribution was assessed with the Shapiro–Wilk normality test, and differences in medians were tested with the Mann–Whitney U test. The categorical variables were compared with the two-tailed Fisher’s exact test. The z scores of RTL were calculated from the mean and SD of the HS group, used as a reference point. The distribution between more than two groups was assessed with the Kruskal–Wallis test. The significance level was set at *p* < 0.05. Statistical analyses were performed using Statistica 13.1 (TBICO Software, Palo Alto, CA, USA) and JASP 0.10.2 (University of Amsterdam, Amsterdam, The Netherlands).

## 3. Results

The basic characteristic of the groups studied are presented in Table 1.

### 3.1. Telomere Length Assessment

**Figure 1:**
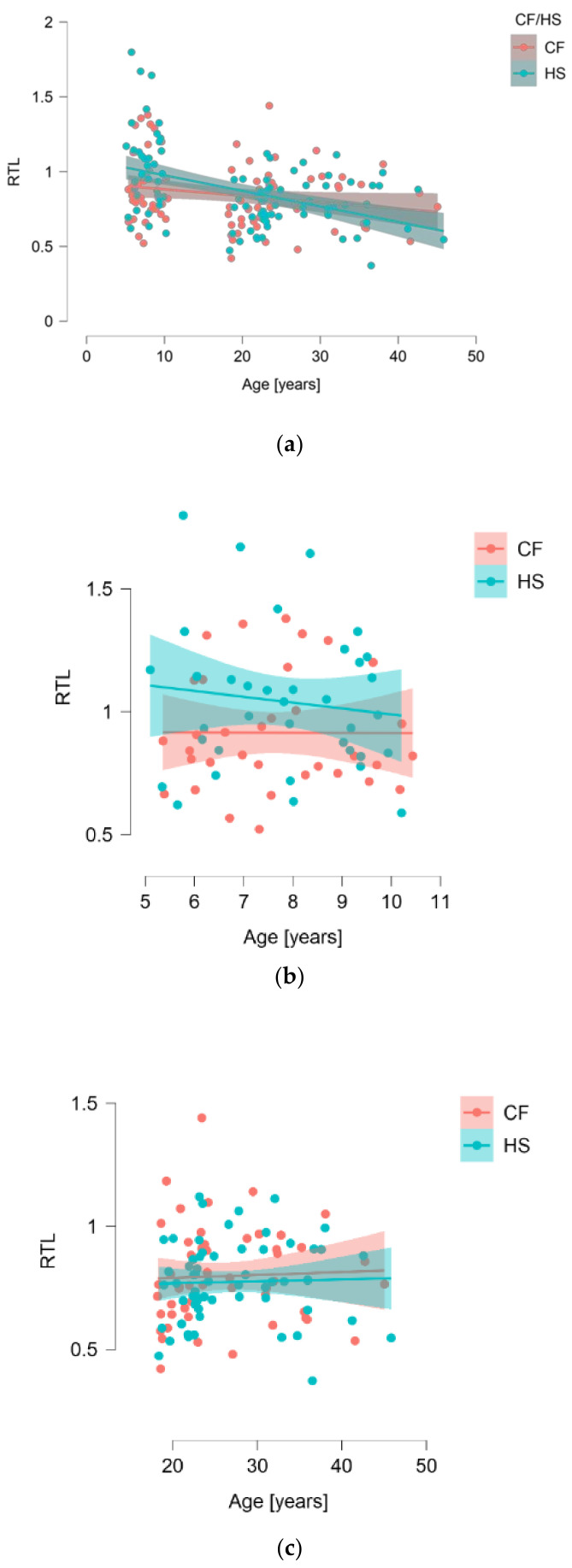
Correlation of relative telomere length (RTL) with age for the whole group studied (**a**), children (**b**), and adults (**c**).

With regard to our complete dataset of 173 individuals, we observed a significant correlation between RTL and age (*p* = 0.0084) (Figure 1a), with shorter telomeres occurring in older subjects. However, when assessing the correlation between telomere length and CF patients (n = 86) and HS (n = 97), there was no difference (median (IQR): 0.799 (0.686–0.950) vs. 0.831 (0.707–0.986); *p* = 0.5283) (Figure 1). This was also true whenever separating subjects into adults (*p* = 0.7188) and children (*p* = 0.0609) (Figure 1).

Overall, females had similar telomere length to males (median (IQR): 0.837 (0.722–0.973) vs. 0.787 (0.641–0.960); *p* = 0.1791), a fact consistent in children exclusively, where RTL of girls did not differ from boys (*p* = 0.5255). However, in the group of adults, women had significantly longer telomeres than men (median (IQR): 0.805 (0.715–0.931) vs. 0.703 (0.574–0.790); *p* = 0.0002). This difference was true for CF (median (IQR): 0.805 (0.715–0.931) vs. 0.709 (0.574–0.790), *p* = 0.0243) as well as HS (median (IQR): 0.804 (0.726–0.906) vs. 0.664 (0.560–0.753), *p* = 0.0023). When separated into sex groups, RTL did not differ between females with CF and healthy females (*p* = 0.4446), nor males with CF and healthy males (*p* = 0.9174) either.

### 3.2. Clinical Expression and Selected Measures of Disease Severity

In order to assess the effects of various clinical parameters (Table 2) on telomere length, we focused on the 87 CF patients exclusively. Seventy-four (85.1%) patients were pancreatic insufficient (Table 2). Median elastase-1 measurement (IQR) was 15 (15–50) μg/g (n = 33). After analyzing the data for children and adult cohorts separately we found that lung involvement in children was predominantly mild/moderate (FEV_1_% median (IQR): 92 (79–104)%), and moderate/severe in adults (FEV_1_% median (IQR): 44 (30–61)%). Twenty of the CF-subjects (23.0%) were homozygous and 48 (55.2%) were heterozygous for the most common mutation in CFTR: F508del (deletion of phenylalanine 508). The remaining twelve patients (13.8%) had other mutations (Table S1 (Supplementary Materials)). None of the children had liver cirrhosis or CF-related diabetes. Out of the parameters investigated only a handful revealed a statistically significant association to telomere length, and in particular those involving different treatments.

Subjects being treated with antibiotics (55.2%) had shorter telomeres than non-treated (*p* = 0.0172). The same pattern was observed for the treatment with inhaled corticosteroids (49.4% of the CF patients), where those being treated had a significantly shorter RTL (*p* = 0.0007). More specifically, a multiple linear regression model with the backward predictor entry using the covariates: gender, age, BMI, child/adult status, and inhaled corticosteroids treatment showed that only inhaled corticosteroids associated with RTL F (1, 64) = 14.64 *p* = 0.0003. 

Furthermore, patients with comorbidities had shorter telomeres, a pattern that was confirmed in a multiple linear regression analysis after adjustment for age, gender, BMI, child/adult status, inhaled corticosteroids, and antibiotics treatment RTL F (1, 60) = 13.65 *p* = 0.0005.

**Figure 2:**
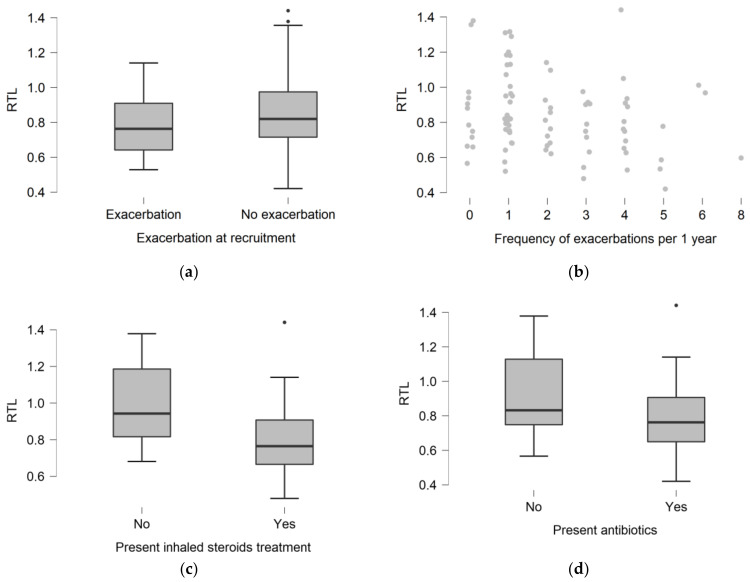
The distribution of relative telomere length (RTL) depending on presence of pulmonary exacerbation at the moment of recruitment (**a**). The distribution of telomere length depending on the frequency of pulmonary exacerbations per year of the disease (**b**). The distribution of telomere length depending on the presence of inhaled steroids (**c**), and antibiotics (**d**) treatment.

To investigate the association of telomere length with lung involvement severity, we correlated RTL and FEV_1_%, and found no relationship (Spearman’s rho = 0.158; *p* = 0.1725). RTL was not related to CFTR mutation class severity (*p* = 0.2816) (Table 2). Twenty-one (24.1%) patients suffered from pulmonary exacerbations at the time of recruitment to the study. Patients with exacerbations did not differ in RTL compared to patients without exacerbations (0.763 (0.642–0.910) vs. 0.820 (0.715–0.975); *p* = 0.1303) (Figure 2a). The RTL value did not differ depending on the frequency of exacerbations (*p* = 0.0550) (Figure 2b). The number of exacerbations did not correlate significantly with RTL (rho = −0.215; *p* = 0.0512).

**Figure 3:**
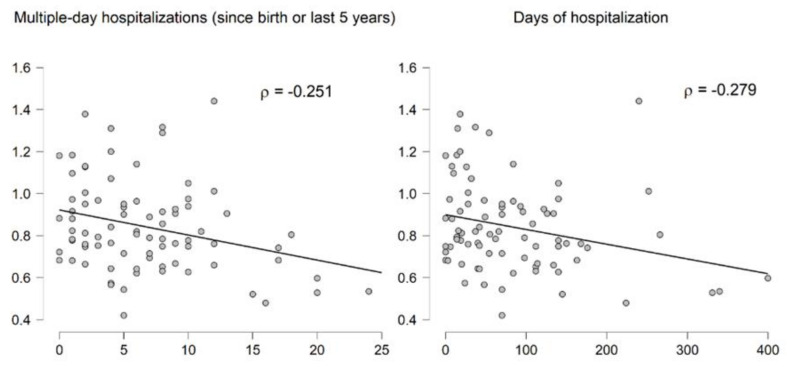
Correlation of relative telomere length (RTL) with hospitalization modalities.

Lastly, to investigate whether RTL was associated with disease severity in CF, we used estimated numbers of hospitalizations and days spent in hospital. RTL correlated with the number of multiple-day hospitalizations (rho = −0.251; *p* = 0.0239), as well as the number of days spent in hospital (rho = −0.279; *p* = 0.0113) (Figure 3). Multiple regression analysis adjusted for age, gender, BMI, frequency of exacerbations, and child/adult status showed that RTL was significantly associated with the number of multiple-day hospitalizations F (2, 75) = 5.49, *p* = 0.0060.

Children with CF treated with inhaled corticosteroids had shorter RTL compared with those not treated (median (IQR): 0.753 (0.627–0.829) vs. 1.045 (0.857–1.237); *p* = 0.0010). This difference was not found in the adult cohort (*p* = 0.8265). Children with comorbidities had shorter RTL than those without comorbidities (median (IQR): 0.785 (0.664–0.881) vs. 0.989 (0.794–1.200); *p* = 0.0045). This difference was absent in the adults (*p* = 0.1590).

## 4. Discussion

In our study, we measured RTL in well-characterized cohorts of CF patients and healthy counterparts, both children and adults. We did not observe any significant differences in RTL between CF and HS, irrespective of the age category of the subjects, but we found a significantly longer RTL in women compared with men overall, which was also confirmed in the CF and HS groups separately. In addition, we observed shorter telomeres in patients treated with inhaled corticosteroids, and independently with antibiotics. Treatment with inhaled corticosteroids remained significantly associated with RTL, even after adjustment for age, gender, BMI, and age category. Shorter telomeres were also independently associated with the presence of CF comorbidities.

Previous data did not show a difference in lung telomere length between CF patients (n = 12) and healthy donors (n = 13) [27]. Another study on airway epithelial cells in CF subjects (n = 18) and controls (n = 18) also did not show differences [28]. Although no RTL difference between CF and HS was identified herein, inhaled corticosteroids associated with shorter RTL. Athanasoulia-Kaspar et al. reported that patients with non-functioning pituitary adenomas and higher daily cortisol doses had shorter telomeres [29]. Furthermore, an in vitro study showed a considerable reduction (50%) in telomerase activity in T-lymphocytes after exposure to high cortisol levels [30]. Inhaled corticosteroids are known for their ability to decrease the influx of T lymphocytes, eosinophils, and mast cells, and inhibit NF-κB activity, chemotaxis, and the synthesis of multiple proinflammatory mediators [31]. This suggests that inhaled corticosteroids influence RTL by altering the composition of leukocytes.

Another treatment associated with shorter RTL in our study was the use of antibiotics. Since the dose, type, and mode of administration were variable in our cohort, we focused on the overall use of antibiotic treatment. Little is known about the influence of antibiotics on telomere length, and evidence is only available for compounds that are not used in clinical practice, such as rubromycins, which are potent human telomerase inhibitors [32,33]. Helby et al. found that in over 75,000 individuals from the general population and 23 years of follow up infections in general, and pneumonia in particular, associated with shorter leukocyte telomere length [34]. It cannot be excluded that the telomere length is associated with the immunocompetence of the host. As previously shown in IPF patients, shorter telomere length identified risk for adverse clinical outcomes in patients exposed to immunosuppressive medications, but there are no data to date regarding the use of antibiotics [35]. Together with our findings, this suggests that the relationship between infections, antibiotics, and telomere length warrants further research.

Telomeres have long been known to be shorter in men. However, the difference was not universally found across different methods of measurement [36]. A meta-analysis showed that, on average, females have longer telomeres than males, which is likely linked to sex hormones [37,38]. We observed a significant difference between genders exclusively in the adult cohort, and the lack of this correlation within children is in line with previous findings in cohorts of 160–800 children that reported no differences between sexes [39,40]. However, in a study with over 4000 children aged 4, girls had longer telomeres than boys, even after adjustment for ethnicity and maternal age [41]. Therefore, sample size seems to be the key factor in the studies of the minute RTL differences between boys and girls. Intriguingly, the method of RTL measurement also seems to play a role, as indicative in the meta-analysis of Gardner et al., which suggests that the Southern blot technique is more powerful in detecting telomere length variations [36]. Taking into account the sample size, the gender-related differences identified in our study are consistent with the literature.

To limit the invasiveness of sample collection, we used dried blood spots, which could easily be employed as an accessible source material for clinical research. Although we found correlations between RTL and disease severity measures, their strength and clinical utility remain unclear. We found that RTL was associated with the number of hospitalizations and days spent in a hospital, which might suggest that telomere length in CF could be ascribed to disease severity. We argue that the duration of hospitalization could be indicative of the severity of the disease, and hence telomere length in CF might indirectly weakly reflect disease severity. The relationship between inhaled steroids and RTL warrants further research, which could employ cytometric characterization, sorting of leukocyte subsets, and assessment of their RTL, and with potential implications for other diseases, such as asthma. The cross-sectional design of the present study makes it difficult to draw causality, and cannot fully explain the direction of cause and effect.

## 5. Conclusions

In conclusion, leukocyte RTL in children and adults with CF is not different than in healthy controls, and does not seem to have any potential as a predictor of CF survival. Shorter telomeres in CF were independently associated with the presence of comorbidities, and treatment with antibiotics and inhaled corticosteroids, as well as the summary hospitalization length. Gender-related differences were present in adult CF patients and HS. Our results indicate that RTL is more strongly associated with specific CF characteristics than with the disease itself.

## Figures and Tables

**Table 1:** Characteristics of children and adults participating in the study.

Parameter Median (IQR) Children Aged 5–10	CF n = 34	HS n = 36	*p* Value
Age, years	7.5 (6.3–8.7)	7.9 (6.5–9.2)	0.587
Sex ratio F/M	15/19 (44%)	14/22 (39%)	0.836
Body weight, kg	23.5 (21.9–27.3)	26.5 (22.3–32.5)	0.059
Body height, cm	123.0 (118.0–133.0)	127.0 (122.5–136.0)	0.095
BMI ^1^, kg/m^2^	15.5 (14.9–16.6)	16.2 (15.5–17.5)	**0.048**
Body weight, z score	−0.627 (−0.846–0.069)	−0.057 (−0.567–0.632)	**0.020**
Body height, z score	−0.435 (−1.154–0.129)	0.061 (−0.604–0.838)	**0.045**
BMI, z score	−0.386 (−0.809–0.144)	−0.040 (−0.445–0.459)	**0.048**
RTL ^2^, z score	−0.511 (−0.922–0.216)	−0.210 (−0.689–0.508)	0.159
**Parameter Median [IQR] Adults Aged 18–45**	**CF n = 53**	**HS n = 61**	***p* Value**
Age, years	23.5 (20.8–31.1)	23.6 (22.5–31.7)	0.244
Sex ratio F/M	27/26 (50.9%)	40/21 (65.6%)	0.114
Body weight, kg	53.8 (48.5–62.0)	60.0 (55.0–76.0)	**<0.001**
Body height, cm	168 (163–175)	170 (164–178)	0.188
BMI, kg/m^2^	19.1 (17.9–22.2)	21.6 (19.8–23.8)	**<0.001**
RTL, z-score	−0.06 (−0.61–0.94)	−0.03 (−0.51–0.67)	0.792

^1^ BMI—body mass index. ^2^ RTL—relative telomere length. Bold indicates statistically significant results (*p* < 0.05).

**Table 2:** Clinical characteristics of patients with cystic fibrosis (children and adults).

Variables	N (%)	Relative Telomere Length Median [IQR]		*p* Value
		Yes	No	
Liver disease	66 (75.9)	0.806 (0.694–0.950)	0.780 (0.651–0.943)	0.6566
Liver cirrhosis	5 (5.7)	0.812 (0.709–0.910)	0.794 (0.683–0.964)	0.6314
Diabetes	12 (13.8)	0.879 (0.696–0.972)	0.792 (0.683–0.950)	0.7935
Nasal polyps	10 (11.5)	0.844 (0.749–0.910)	0.786 (0.682–0.966)	0.9940
Chronic sinusitis	50 (57.5)	0.799 (0.683–0.950)	0.807 (0.715–1.005)	0.5647
Meconium ileus	7 (8.0)	0.631 (0.574–1.130)	0.809 (0.715–0.950)	0.2530
DIOS *	2 (3.8)	0.744 (0.586–0.901)	0.761 (0.652–0.914)	0.6800
Prevalence of asthma	3 (3.4)	0.760 (0.714–0.833)	0.805 (0.689–0.957)	0.6294
Pancreatic insufficiency	74 (85.1)	0.799 (0.683–0.950)	0.821 (0.729–0.943)	0.7459
Children’s FEV_1_% ≤ median	13 (52.0)	0.694 (0.621–0.765)	0.784 (0.707–0.904)	0.1683
Adults’ FEV_1_% ≤ median	26 (51.0)	0.763 (0.603–0.904)	0.763 (0.715–0.927)	0.2828
Chronic or intermittent *Pseudomonas aeruginosa* infection	44 (50.6)	0.762 (0.660–0.908)	0.824 (0.742–1.097)	0.0569
F508del homozygote	20 (23.0)	0.803 (0.674–0.957)	0.805 (0.683–0.968)	0.8272
F508del heterozygote	48 (55.2)	0.787 (0.688–0.939)	0.881 (0.683–0.973)	0.3662
Other mutations	12 (13.8)	0.908 (0.755–1.022)	0.792 (0.681–0.950)	0.2032
**Mutation class severity**				0.2869
mild/-	2 (2.5)	0.989 (0.905–1.072)		
severe/-	11 (13.6)	0.742 (0.660–0.820)		
severe/mild	7 (8.6)	0.881 (0.749–1.357)		
severe/severe	61 (75.3)	0.812 (0.683–0.973)		
**Treatment**				
Present antibiotics	48 (55.2)	0.762 (0.648–0.908)	0.832 (0.748–1.129)	**0.0172**
Antibiotics in past 6–12 months *	23 (43.4)	0.749 (0.643–0.910)	0.805 (0.667–0.927)	0.3795
Present inhaled steroids treatment	43 (49.4)	0.765 (0.664–0.910)	0.943 (0.813–1.191)	**0.0007**
Inhaled steroids treatment in past 6–12 months *	19 (35.8)	0.749 (0.643–0.910)	0.901 (0.747–1.012)	0.0829
Present beta-mimetics	79 (90.8)	0.794 (0.681–0.964)	0.935 (0.785–0.940)	0.2812
Beta-mimetics in past 6–12 months *	24 (45.3)	0.749 (0.637–0.861)	0.831 (0.667–0.950)	0.3080
Present digestive medications ***	59 (67.8)	0.805 (0.667–0.950)	0.885 (0.771–0.954)	0.4450
Digestive medications in past 6–12 months */***	14 (26.4)	0.749 (0.627–0.910)	0.805 (0.667–0.927)	0.5238
Present probiotics *	32 (60.4)	0.761 (0.624–0.908)	0.883 (0.747–0.889)	0.3624
Probiotics in past 6–12 months *	13 (24.5)	0.769 (0.643–0.910)	0.761 (0.634–0.903)	0.9366
Other medications ****	85 (97.7)	0.783 (0.683–0.940)	0.778 (0.689–0.911)	0.4699
Comorbidities **	55 (63.2)	0.763 (0.643–0.905)	0.950 (0.783–1.130)	**0.0006**

* data available only for adult cohort. ** e.g., hypothyroidism, asthma, osteopenia, osteoporosis, acute pancreatitis, cholecystolithiasis, hypogammaglobulinemia, allergy. *** e.g., omeprazole. **** other than ursodeoxycholic acid, ornithine, phospholipid, Silybum marianum fruit extract. Bold indicates statistically significant results (*p* < 0.05).

## Data Availability

Data available on request due to restrictions e.g., privacy or ethical.

## Supplementary Materials

The following are available online at https://www.mdpi.com/2077-0383/10/4/590/s1. Table S1: Genotype frequencies in adults and children with cystic fibrosis.
