# Supplementary material for: Leukocyte Telomere Length Is Not Reduced in Children and Adults with Cystic Fibrosis but Associates with Clinical Characteristics—A Cross-Sectional Study
Source: J Clin Med. 2021 Feb 4;10(4):590. doi: 10.3390/jcm10040590 (PMC7915028; doi:10.3390/jcm10040590)
Supplement: Supplementary file 1 [file jcm-10-00590-s001.pdf]

Table S1. Genotype frequencies in adults and children with cystic fibrosis.

Genotype Frequency in Children	Frequency	Percent	Genotype Frequency in Adults	Frequency	Percent
2184insA/2789 + 2insA	1	2.9	F508del/2143delT	2	4.1
F508del/-	2	5.9	F508del/2183AA > G	3	6.1
F508del/1717-1G > A	2	5.9	F508del/3849 + 10kbC > T	3	6.1
F508del/2143delT	1	2.9	F508del/F508del	14	28.6
F508del/2183AA > G	1	2.9	F508del/del2,3(21kb)	4	8.2
F508del/2184insA	2	5.9	F508del/1717-1G > A	3	6.1
F508del/296 + 1G > T	1	2.9	F508del/-	2	4.1
F508del/3849 + 10kbC > T	1	2.9	3272-26A > G	1	2
F508del/E92K	1	2.9	F508del/1078delT	1	2
F508del/F508del	8	23.5	F508del/1898 + 1G > c	1	2
F508del/G542X	1	2.9	F508del/2184insA	1	2
F508del/G85E	1	2.9	F508del/2721del	1	2
F508del/R1102X	1	2.9	F508del/3171insC	1	2
F508del/R347P	1	2.9	F508del/N1303K	1	2
F508del/R553X	1	2.9	F508del/R334W	1	2
F508del/T582I	1	2.9	F508del/R851X	1	2
F508del/c.53 + 2T > C	1	2.9	F508del/W1282X	1	2
F508del/dele2,3(21kb)	3	8.8	G27V/F508del	1	2
G524X/G524X	1	2.9	Q1281X/W1204X	1	2
G542X/N1303K	1	2.9	c.302T > G3718-2477C/2002C > T	1	2
R553X/3849 + 10kbC > T	1	2.9	del2,3(21kb)/cftr12m	1	2
W1282X/dele2,3(21kb)	1	2.9	1717-1G > A/2183AA > G	1	2
			del2,3(21kb)/-	1	2
			del2,3(21kb)/del2,3(21kb)	1	2
